# Degradation pathways in standard and inverted DBP-C_70_ based organic solar cells

**DOI:** 10.1038/s41598-019-40541-6

**Published:** 2019-03-11

**Authors:** Golnaz Sherafatipour, Johannes Benduhn, Bhushan R. Patil, Mehrad Ahmadpour, Donato Spoltore, Horst-Günter Rubahn, Koen Vandewal, Morten Madsen

**Affiliations:** 1 SDU NanoSYD, Mads Clausen Institute, University of Southern Denmark, Sønderborg, Denmark; 2 Dresden Integrated Center for Applied Physics and Photonic Materials (IAPP) and Institute for Applied Physics Technische Universität Dresden Nöthnitzer Str. 61, 01187 Dresden, Germany; 3 Present Address: Institute for Materials Research (IMO-IMOMEC), Hasselt University, Wetenschapspark 1, 3590 Diepenbeek, Belgium

## Abstract

Achieving long-term stability in organic solar cells is a remaining bottleneck for the commercialization of this otherwise highly appealing technology. In this work, we study the performance and stability differences in standard and inverted DBP/C_70_ based organic solar cells. Differences in the charge-transfer state properties of inverted and standard configuration DBP/C_70_ solar cells are revealed by sensitive external quantum efficiency measurements, leading to differences in the open-circuit voltages of the devices. The degradation of standard and inverted solar cell configurations at ISOS aging test conditions (ISOS-D-3 and ISOS-T-3) was investigated and compared. The results indicate that the performance drop in the small molecule bilayer solar cells is less related to changes at the D-A interface, suggesting also a pronounced morphological stability, and instead, in the case of inverted cells, dominated by degradation at the electron transport layer (ETL) bathocuproine (BCP). Photoluminescence measurements, electron-only-device characteristics, and stability measurements show improved exciton blocking, electron transport properties and a higher stability for BCP/Ag ETL stacks, giving rise to inverted devices with enhanced performance and device stability.

## Introduction

During the past years, organic solar cells (OSCs) have received a great deal of attention as they offer unique advantages such as low fabrication cost, semi-transparency and lightweight modules. Moreover, the power conversion efficiency (PCE) of OSCs recently reached up to 14.57%^1^ and 17.3%^2^ for single junction and tandem cells, respectively, showing the great potential of the technology. However, to be commercially viable, OSCs need to obtain long-term stabilities of at least 10 years^3,4^, which has presented a significant challenge to this otherwise promising technology. Therefore, a deeper insight into the degradation mechanisms of the devices is needed.

In general, degradation mechanisms of OSCs can be divided into two main categories: extrinsic and intrinsic degradation. The former is caused by diffusion of water and oxygen into device layers, and the latter is due to the intrinsic nature of organic materials and involved interlayers, and includes undesirable chemical changes as well as molecular rearrangements at the device interfaces and within the active materials^5^. Encapsulation of the OSCs can significantly slow down the extrinsic degradation, while intrinsic degradation can occur even for perfectly encapsulated devices^6–13^. Relevant factors include electrode diffusion and morphological changes, resulting in a complex range of degradation mechanisms, which imply that the interface between every two adjacent layers has a strong impact on the stability of the devices^14^. In organic solar cells, one of the critical places for intrinsic degradation is the interface between the electron donating (D) and accepting (A) material where chemical and morphological changes over time directly affect photogeneration of charge carriers and recombination processes. Residing at the D-A interface, the charge-transfer (CT) state mediates the charge carrier separation and the free charge carrier recombination, and its properties directly affect the device performance, especially the open-circuit voltage (V_OC_)^15–17^. Furthermore, degradation paths are rather complex and differ between standard and inverted stack architectures^18–21^. For example, Krebs *et al*. and Cros *et al*. reported that for the two types of architectures, with the same type of photoactive layer and identical processing procedure, the inverted configuration devices show a higher air stability^22,23^. This however depends on the exact type of layer stack used, and cannot be used as a general rule. Specific interlayers may also lead to different stability trends for standard and inverted device architectures. For example, the hole transport layer molybdenum oxide has been shown to lead to more stable device performance than PEDOT:PSS hole transport layers in standard configuration devices^24^, whereas the exact opposite is the case in inverted configuration cells^25^. This sets the need for an individual lifetime assessment of any new layer stack embedded in OPV technology.

In this work, we investigate degradation pathways in Tetraphenyldibenzoperiflanthen (DBP) and Fullerene (C_70_) based organic solar cells, having standard and inverted device architectures. DBP is a promising electron donor material, which has been utilized in organic solar cells since 2009^26–34^. Its advantages, such as high optical absorption strength and deep highest occupied molecular orbital (HOMO) level around 5.5 eV makes it a good match to fullerene acceptors, resulting in reasonably high open-circuit voltages^31,35–39^. We use sensitive external quantum efficiency (sEQE) measurements to detect morphological differences between standard and inverted device configurations, and to detect potential degradation at the DBP/C_70_ interface after aging the devices at ISOS-D-3 (darkness, 80 °C, 85% relative humidity (RH)) and ISOS-T-3 (darkness, −40 °C, ambient humidity) aging conditions. Our findings show that despite the changes in V_OC_ values of the fresh and degraded devices, CT state properties undergo only very minor changes, suggesting a pronounced morphological stability at the DBP/C_70_ interface. Instead, it is demonstrated that the inverted devices suffer from higher V_OC_ losses and low stability due to the use of a Bathocuproine (BCP) electron transport layer (ETL)^40^. To address this challenge, we introduce a BCP/Ag stack as an alternative ETL with improved carrier transport efficiency and exciton blocking properties. Our final results show that inverted devices implementing BCP/Ag stacks as ETL lead to enhanced device stabilities.

## Results and Discussion

**Figure 1:**
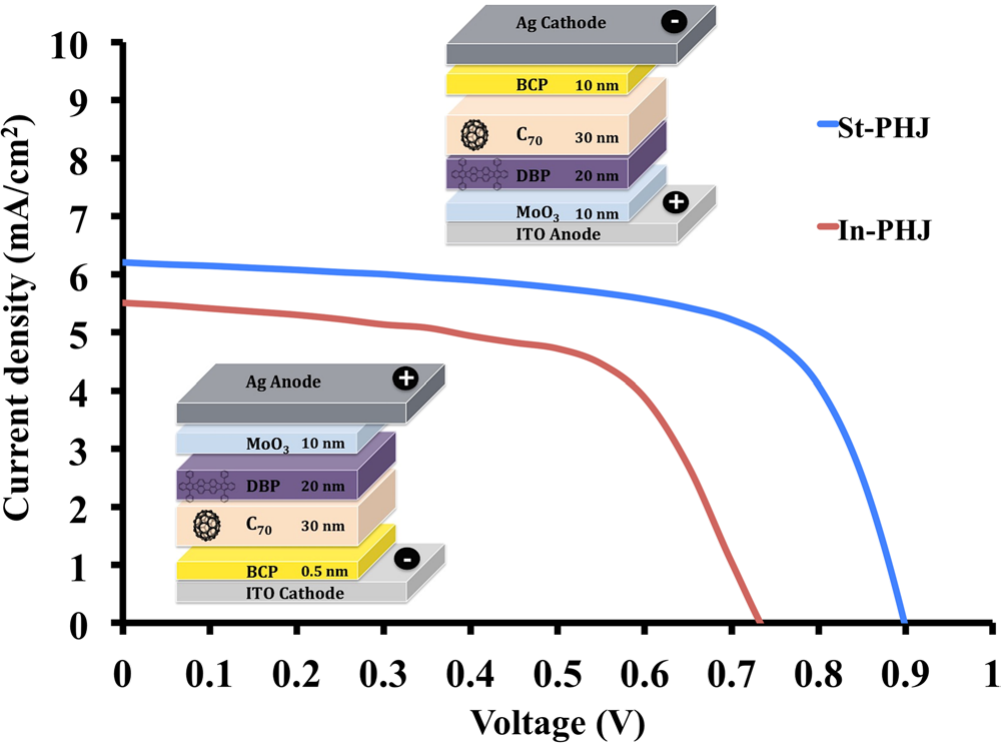
J-V scans of DBP/C_70_ organic based planar heterojunction (PHJ) devices having standard (St-PHJ) and inverted (In-PHJ) device configurations.

The J-V characteristics of the standard and inverted devices with identical contacts and active layers are presented in Fig. 1 and Table 1. The parameters, shown in Table 1, are averaged over 7 devices of each type, and J-V curves in Fig. 1 show devices with characteristics closest to the average parameters.

**Table 1:** OSC performance parameters of DBP/C_70_ organic based PHJ devices having standard (St-PHJ) and inverted (In-PHJ) device configurations.

Device type	V_OC_ (V)	J_SC_ (mA/cm^2^)	PCE (%)	FF (%)
St-PHJ	0.85 ± 0.05	6.32 ± 0.36	3.66 ± 0.21	68.0 ± 1.8
In-PHJ	0.72 ± 0.07	5.78 ± 0.40	2.68 ± 0.29	62.0 ± 2.5

The reported values are averaged from the extracted measurements of total 7 devices of each type.

Although both device types have identical active layers, the devices based on the standard structure show an overall better performance, reaching 130 mV higher V_OC_ and higher PCE. Recent work has demonstrated that this trend, however, may depend on device area^41^. We note that the reported performances here are similar to those reported for bilayer DBP/C_70_ cells in the literature^32,42^.

**Figure 2:**
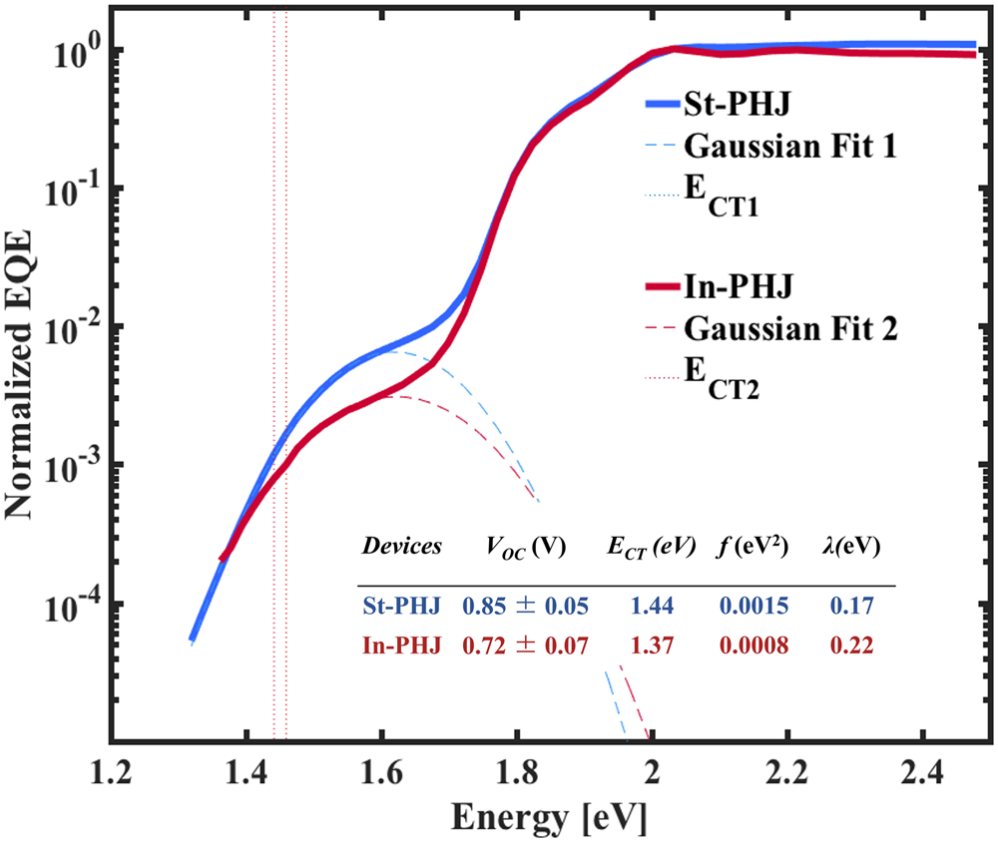
sEQE measurements and Marcus fits for standard and inverted solar cells (solid lines) as a function of the photon energy. Dashed lines show Gaussian fits to the low energy part of the sEQE spectrum, using Marcus theory.

Previous studies have shown that morphological differences and changing molecular orientation at the D-A interface can lead to V_OC_ changes by altering the material energy levels^43^ and the properties of the CT states. We investigate the differences in V_OC_ of the devices through the sEQE measurements and CT state characteristics. Based on the electron transfer theory developed by Marcus^44,45^, energy of the CT state (E_CT_), the reorganization energy (λ), and the amplitude of the CT absorption band (*f)* are determined by fitting the low energy part of the EQE spectrum with a Gaussian, using the method outlined in reference^15^. We obtain that E_CT_ is 1.44 eV for the standard and 1.37 eV for the inverted device configuration. The normalized sEQE spectra and their corresponding fits and extracted CT state parameters are shown in Fig. 2.

**Figure 3:**
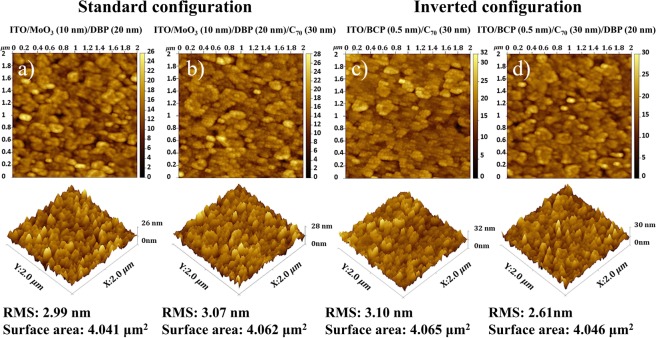
AFM images of interface layer (**a**) ITO/MoO_3_ (10 nm)/DBP (20 nm), (**b**) ITO/MoO_3_/DBP (20 nm)/C_70_ (30 nm), (**c**) ITO/BCP (0.5 nm)/C_70_ (30 nm), and (**d**) ITO/BCP (0.5 nm)/C_70_ (30 nm)/DBP (20 nm).

The 70 meV higher E_CT_ in the standard configuration is in agreement with the higher V_OC_ value obtained, although it does not explain the full change in V_OC_ upon inverting the structure. A slightly higher *f* value is also obtained for the standard configuration, which identifies as a higher density of CT states, being correlated to a higher amount of interface between the donor and acceptor molecules^17^. Previous findings indicate that a larger interface area results in a higher recombination current^46^; however, the difference in *f* value seen here is minor, and should not contribute to significant V_OC_ differences. Atomic force microscopy (AFM) images were recorded for the top layers in (a) ITO/MoO_3_/DBP, (b) ITO/MoO_3_/DBP/C_70_, (c) ITO/BCP/C_70,_ and (d) ITO/BCP/C_70_/DBP stacks, as shown in Fig. 3, in order to investigate further the morphology at the interface. The first two and the second two stacks represent the layers in standard and inverted configurations, respectively. The root-mean-square (RMS) roughness and surface area values were extracted and are shown in Fig. 3.

Figure 3a,c show the surfaces of the lower layer of the D-A interface in each type of device. In the standard configuration (Fig. 3a), the DBP layer has a slightly smoother surface with lower surface area, although very close to what is seen for the inverted configuration. This could imply that a non-conformal film coverage for DBP on the C_70_ layer lead to the slightly lower *f* value seen for the inverted device configuration. In general we note that variations in molecular orientations can impact voltage losses, charge generation efficiency, and CT properties^43,47–50^, and that both a modified interface area and modified molecular orientation can lead to the slightly different *f* values observed here.

**Figure 4:**
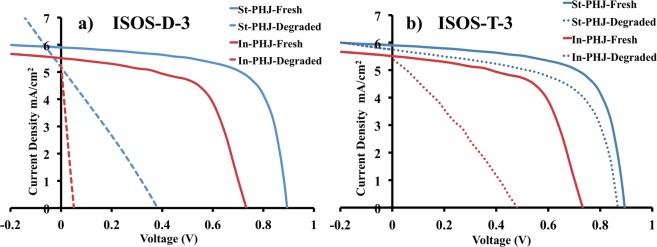
J-V curves for fresh standard (blue) and inverted (red) devices (solid lines) and aged devices (dashed lines) at (**a**) ISOS-D-3 (**b**) and ISOS-T-3 degradation conditions.

Figure 4 shows the J-V characteristics of the fresh cells and cells degraded in ISOS-D-3 and ISOS-T-3 conditions for 24 hours. Average values over 7 devices of each set are presented in Table 2.

**Table 2:** Photovoltaic performance parameters of fresh and aged devices under ISOS-D-3 and ISOS-T-3 degradation conditions.

Devices	V_OC_ (V)	J_SC_ (mA/cm^2^)	PCE (%)	FF (%)
St-Fresh	0.85 ± 0.05	5.8 ± 0.26	3.50 ± 0.17	71.2 ± 1.1
In-Fresh	0.72 ± 0.07	5.88 ± 0.57	2.68 ± 0.29	62.0 ± 2.5
St-ISOS-D-3	0.36 ± 0.28	4.90 ± 1.44	0.68 ± 0.73	23.3 ± 16.7
In-ISOS-D-3	0.03 ± 0.05	5.60 ± 0.54	0.01 ± 0.02	7.3 ± 14.5
St-ISOS-T-3	0.87 ± 0.05	5.67 ± 0.34	2.94 ± 0.52	59.3 ± 6.7
In-ISOS-T-3	0.45 ± 0.07	5.86 ± 0.93	1.02 ± 0.55	37.9 ± 13.8

Device parameters for standard configuration are marked in blue, and for inverted device configuration in red.

Under ISOS-D-3 condition, the PCE values drop significantly for both devices, although it is most significant for inverted cells. The PCE drop is mostly the consequence of a reduction in FF and V_OC_ values, which may be due to an increase in the density of deeper traps caused by oxidation of the active layer^51–53^. These traps can act as recombination centers and disturb the distribution of the internal field^52^. Furthermore, metal diffusion from the top contacts into the buffer layer at the high temperature can also result in a decreased FF^54,55^.

For the ISOS-T-3 condition, the PCE value of the standard devices is less influenced by this aging condition and the V_OC_ value remains intact. This could be due to the slowed down chemical reactions taking place in the organic materials at the low temperatures used. This effect from temperature is demonstrated in the Supplementary Information in Fig. S-1, where degradation of encapsulated standard devices in ISOS-T-3 and ISOS-D-1 (darkness, room temperature) conditions are compared. We observe that for ISOS-T-3 condition, the V_OC_ of the devices remains above 80% of its initial value after 3500 hours. However, for devices kept at room temperature (ISOS-D-1), the T_80_ point is reached much earlier, at around 500 hours^15,56^. However, looking at the results for inverted devices in the same condition (Fig. 4), a notable change in the device stability is seen as compared to the standard configuration cells. Since the same material system is present in both types of devices, this is an indication of a degradation process not related to standard photo-oxidation of the active layer. Under ISOS-D-3 conditions, when humidity and high temperature are included, degradation is accelerated and effects from both chemical reactions in the active layer and instabilities of the interlayers are seen resulting in pronounced degradation.

**Figure 5:**
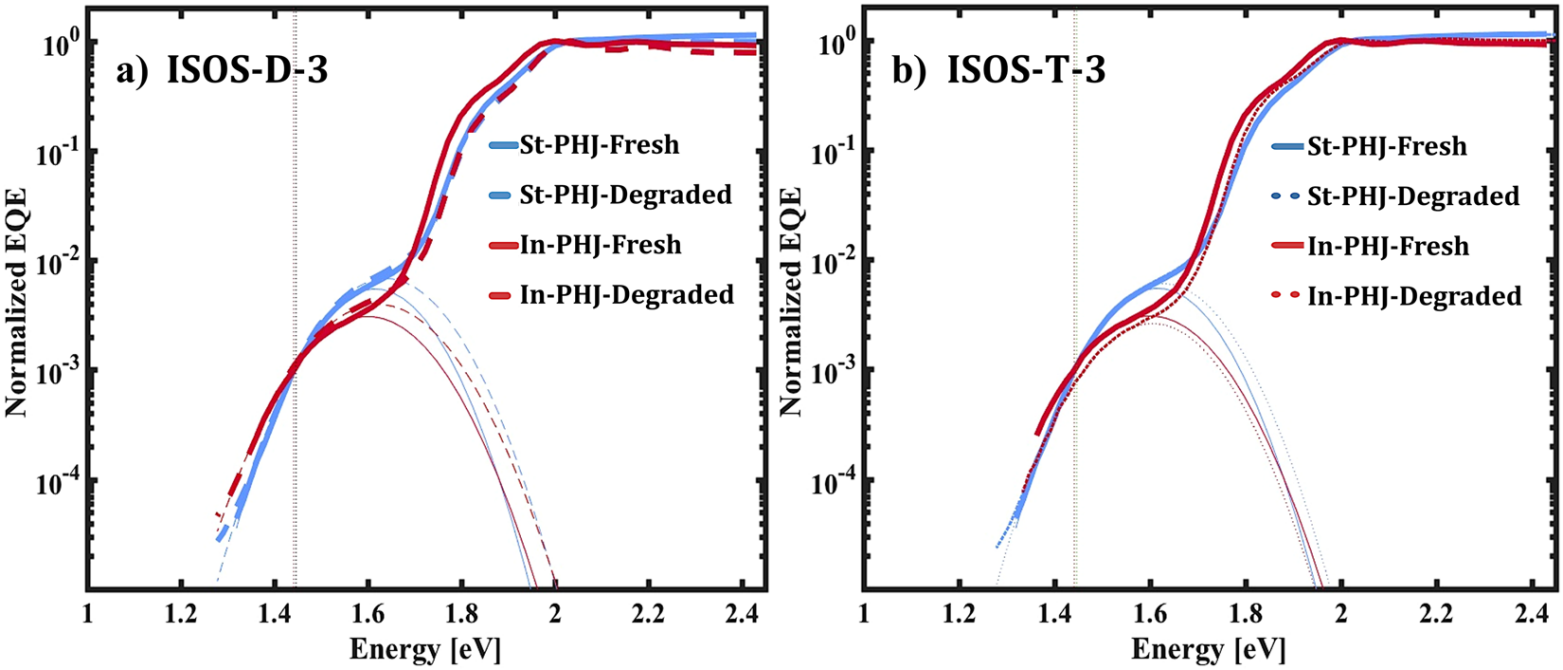
sEQE measurements and Marcus fits for fresh and aged devices at (**a**) ISOS D-3 and (**b**) ISOS-T-3 conditions.

In order to detect possible morphological changes at the D-A interface, sEQE measurements were performed for degraded samples as well. Figure 5 shows normalized sEQE spectra and their corresponding fits for fresh and aged devices at each aging condition. Table 3 presents the extracted CT parameters.

**Table 3:** V_OC_ and CT parameters extracted through fits of sEQE spectra for fresh and aged devices at ISOS-D-3 and ISOS-T-3 degradation conditions.

Devices	V_OC_ (V)	*f* (eV^2^)	λ (eV)	E_CT_ (eV)
St-PHJ-Fresh	0.85 ± 0.05	0.0017	0.17	1.44
In-PHJ-Fresh	0.72 ± 0.07	0.0008	0.22	1.37
St-PHJ-ISOS-D-3	0.36 ± 0.28	0.0017	0.19	1.44
In-PHJ-ISOS-D-3	0.03 ± 0.05	0.0011	0.23	1.38
St-PHJ-ISOS-T-3	0.87 ± 0.05	0.0014	0.18	1.44
In-PHJ-ISOS-T-3	0.45 ± 0.07	0.0006	0.20	1.40

Device parameters for standard configuration are marked in blue, and for inverted device configuration in red.

The sEQE results indicate that although a change in V_OC_ of the devices is measured after degradation, the CT properties remain almost the same. The unchanged *f* value indicates a morphological stability at the DBP/C_70_ interface, suggesting that a drop in voltage upon aging of the devices is not due to morphological changes at the D-A interface. This is furthermore supported by investigations on annealing of standard PHJ and Bulk Heterojunction devices (BHJ), which was conducted at 110 °C for 3 hours inside a glovebox in darkness (see Supplementary Information, Fig. S.2). The results show that although a significantly larger *f* value is seen for the BHJ cells, as expected, no change in CT state energy and *f* value is seen upon degradation of these devices.

For the inverted configuration, the C_70_ absorption (shoulder at 1.8 eV) drops slightly relative to DBP absorption during degradation. We attribute this instability of C_70_ to changes in the underlying BCP layer. It has been shown that BCP can be employed as an ETL in inverted devices, but this can lead to problems in device performance with low FF and PCE^40^, depending on the device area^41^. Moreover, it can give rise to V_OC_ losses at the ETL-C_70_ interface due to its tendency for crystallization and inducing defects^41^.

In the standard device architecture, an Ag-BCP complex is formed due to diffusion of the Ag into the BCP layer^57^. This complex can facilitate electron transport by introducing a new lowest unoccupied molecular orbital (LUMO) level aligned with the LUMO of the C_70_^58^_._ However, the inverted structure lacks this BCP-metal complex and thus efficient electron extraction^59^. For the small device areas of 10 mm^2^ investigated here, reasonable device efficiencies for inverted architectures are observed, but with limited device stability. In order to investigate the instabilities further, we assessed the ETL properties of several thicknesses of BCP and two BCP ETL stacks based on C_70_ and Ag in an inverted configuration. It has been shown that an ultrathin layer of C_70_ between ITO and BCP can improve the ETL properties and the device yield^41^. The second candidate can provide a BCP-Ag semi-complex without directly doping the BCP layer.

**Figure 6:**
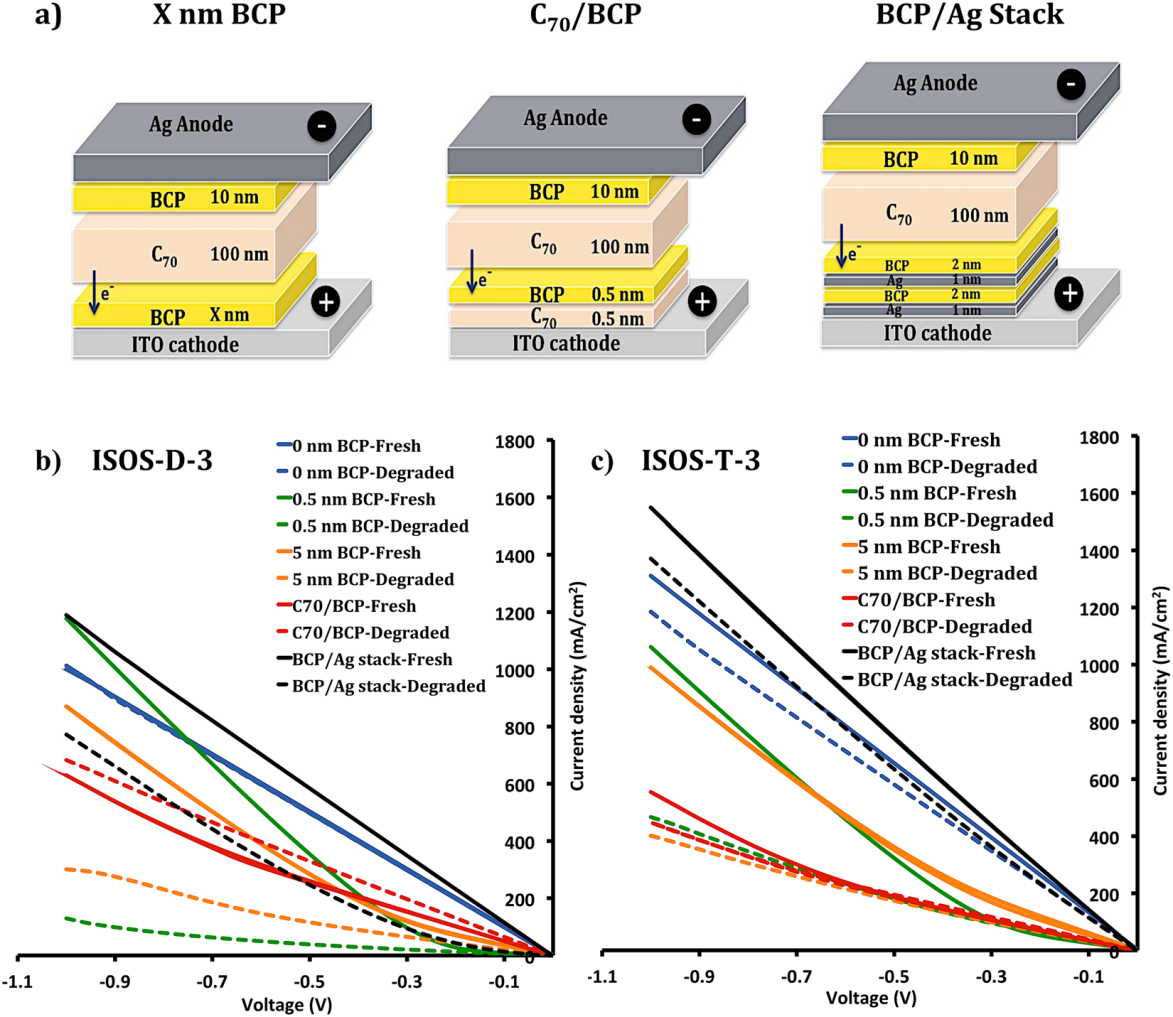
(**a**) Electron-only devices with different ETLs. J-V measurements after aging devices for 24 hours in (**b**) ISOS-D-3 and (**c**) ISOS-T-3 degradation conditions.

Electron transport properties of the implemented ETLs were examined via electron-only devices (EODs) (see Fig. 6a and methods), for both fresh and aged devices. In these devices, electrons are injected into the devices through the Ag electrode and extracted at the ITO side. Figure 6b,c show EOD characteristics after degrading the devices at ISOS-D-3 and T-3 conditions for 24 hours. J-V measurements for the fresh EODs show a lower series resistance for the BCP/Ag stack, indicating better electron transport properties. Interestingly, devices without BCP layer at the bottom (0 nm BCP) show no or very minor degradation, indicating that indeed the BCP layer is responsible for the degradation in the inverted cells. Note that these devices have a top BCP layer, and the results show a high stability for this layer and no major role in the degradation of the devices. The results also show that pure BCP hampers electron extraction in this inverted configuration, as expected due to the lack of a metal-BCP complex.

**Figure 7:**
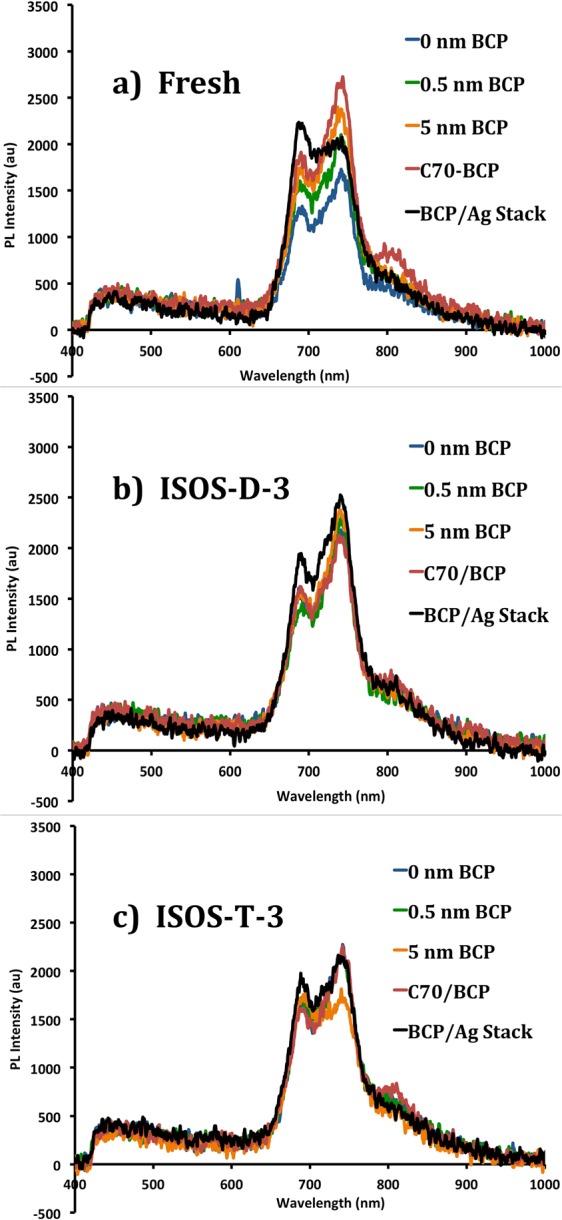
Photoluminescence (PL) measurements for five ETLs, measured “fresh” and degraded at ISOS-D-3 and ISOS-T-3. The BCP/Ag stack has the structure: BCP (2 nm)/Ag (1 nm)/BCP (2 nm)/Ag (1 nm)/BCP (2 nm).

The exciton blocking properties of the ETLs were examined using photoluminescence (PL) measurements. The results presented in Fig. 7a demonstrate that increasing the BCP thickness increases the PL intensity of the C_70_ layer, due to increased exciton blocking of the ETL and thus minimum quenching at the cathode interface.

The stability of these electron transport layers were tested by aging the samples at ISOS-D-3 and ISOS-T-3 conditions for 24 hours, and repeating the PL measurements after the aging process. Figure 7b,c represent the results for each condition. The results show overall lower PL intensities in both standard and inverted configurations comparing degraded to fresh films. However, in each set, the BCP/Ag stack shows better exciton blocking properties with higher PL intensities. From these results we conclude that the BCP/Ag stack both provides improved exciton blocking and electron transport properties, and also less interface degradation. Therefore, we implemented this ETL candidate in full inverted devices, and evaluated the device performance and stability.

**Figure 8:**
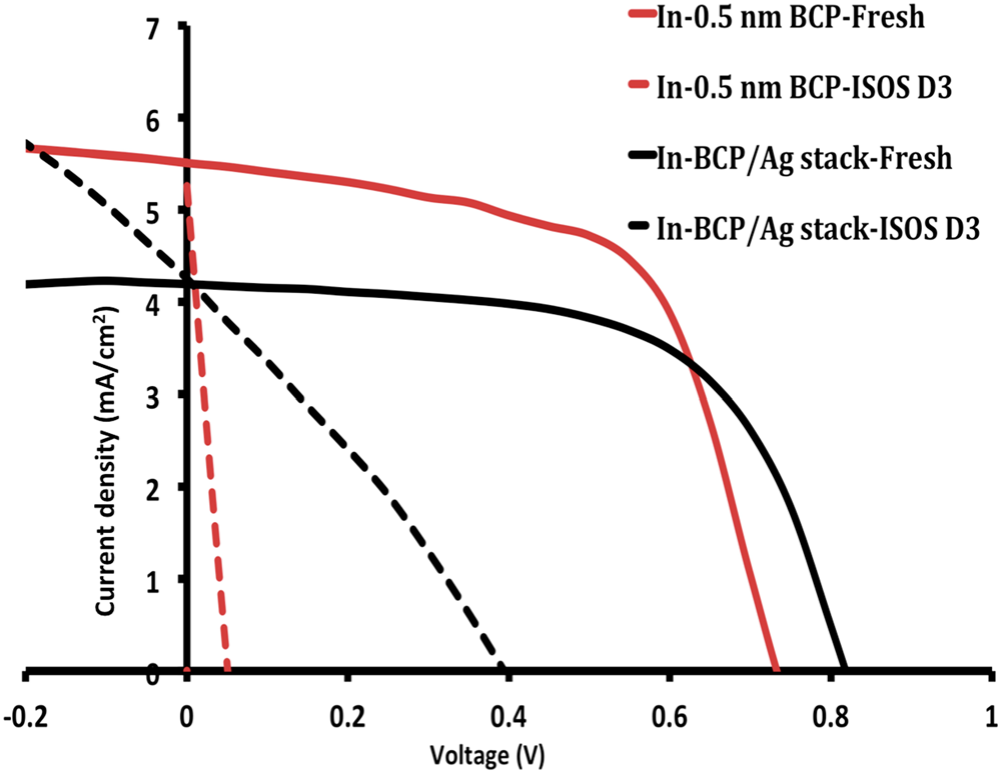
J-V measurements for fresh and aged inverted device with 0.5 nm BCP (ITO/BCP (0.5 nm)/C_70_ (30 nm)/DBP (20 nm)/MoO_3_ (10 nm)/Ag (100 nm)) and BCP/Ag stack (ITO/BCP (2 nm)/Ag (1 nm)/BCP (2 nm)/Ag (1 nm)/BCP (2 nm)/C_70_ (30 nm)/DBP (20 nm)/MoO_3_ (10 nm)/Ag (100 nm)).

Inverted devices with the BCP/Ag stack were fabricated with the structure ITO/(BCP/Ag stack)/C_70_/DBP/MoO_3_/Ag. J-V curves were recorded under 1 sun illumination for fresh and degraded devices in ISOS-D-3 condition, and compared with the previous results for 0.5 nm BCP as ETL. The results are presented in Fig. 8 and Table 4.

**Table 4:** Photovoltaic characteristics of fresh and aged inverted devices with 0.5 nm BCP or BCP/Ag stack as their hole transport layer.

ETL	V_OC_ (V)	J_SC_ (mA/cm^2^)	PCE (%)	FF (%)
0.5 nm BCP-Fresh	0.72 ± 0.07	5.78 ± 0.40	2.68 ± 0.29	62.0 ± 2.5
0.5 nm BCP-ISOS-D-3	0.04	5.26	0.062	26.2
BCP/Ag stack-Fresh	0.82 ± 0.05	4.19 ± 0.16	2.09 ± 0.13	60.4 ± 4.1
BCP/Ag stack-ISOS-D-3	0.37	4.25	0.48	30.2

The obtained results show improved V_OC_ and stability for the BCP/Ag stack ETL based devices, demonstrating that the BCP ETL is causing the initial larger V_OC_ drop for inverted cells, along with the accelerated degradation compared to the standard configuration devices. Since the J-V measurements are performed through a bottom illumination, a slightly lower J_SC_ is resulting from a reflection from the thin layer of Ag at the ETL. Interestingly, the performance of the ISOS-D-3 degraded BCP/Ag based inverted cells show similar performance as the ISOS-D-3 degraded standard configuration cells, indicating that the interlayer now possesses a similar stability as the one implemented in the standard configuration devices. A similar strong improvement in the stability of DBP/C_70_ organic solar cell devices upon improving the molybdenum oxide hole transport layer was recently demonstrated, also pointing to the importance on the stability of the contact layer in such solar cell devices^60^.

In this work, the performance and stability of DBP-C_70_ based organic solar cells in standard and inverted device configurations were studied. Although differences in V_OC_ between standard and inverted devices were observed for fresh cells, the results revealed no morphological or CT state changes at the DBP-C_70_ interface upon degradation in ISOS-D-3 and ISOS-T-3 conditions. Instead, the different ISOS degradation measurements were pointing at electrode or interlayer instabilities being a significant cause of degradation in inverted devices, which was backed up by investigations of the effect of the ETL on device stability. The results demonstrate that this layer is contributing significantly to the observed degradation of the inverted devices. Different BCP film thicknesses and two BCP based ETL stacks were evaluated from PL measurements and electron-only devices. The results show improved performance and a higher device stability for inverted devices based on a BCP/Ag stack as electron transport layer, which thus point to pure BCP being the weak point in the stability of these devices.

## Methods

**Figure 9:**
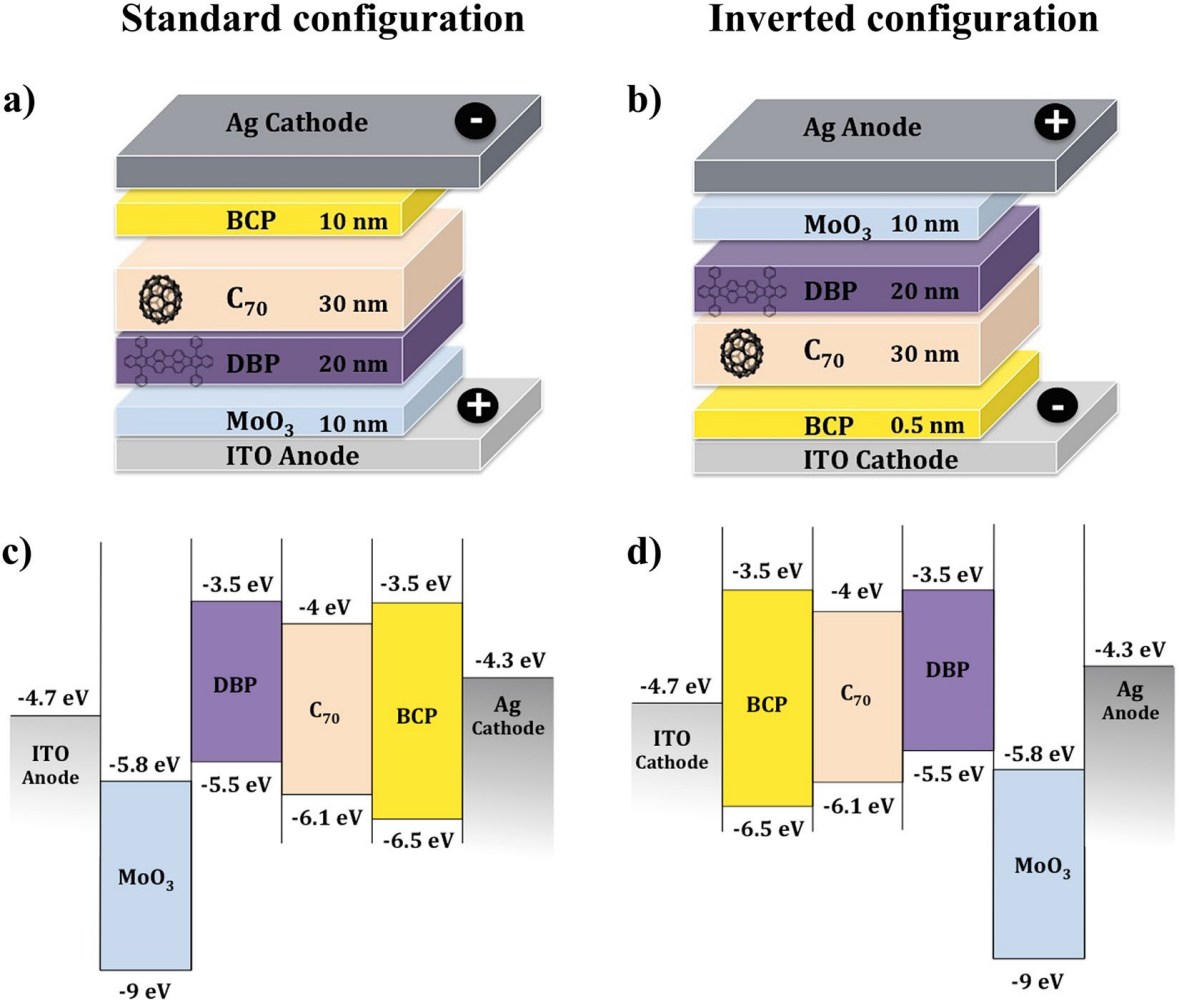
Device architecture of (**a**) standard, and (**b**) inverted structure of the fabricated organic solar cells. Energy level diagram^26,28^ of (**c**) standard and (**d**) inverted DBP/C_70_ based devices.

Solar cells studied in this work were fabricated using DBP as electron donor and C_70_ as electron acceptor. For electron transport and hole transport layers, BCP and molybdenum oxide (MoO_3_) were used, respectively. Silver (Ag) (AESpump ApS, Denmark) was used as top electrode in both types of devices. DBP was purchased from Luminescence Technology Corp., Taiwan, and C_70_, MoO_3_ and BCP were purchased from Sigma-Aldrich, Germany. Solar cell devices were fabricated on pre-coated, pre-patterned indium-tin oxide (ITO) glass substrates with a sheet resistance of 9–15 $\Omega /{sq}$ (purchased from University wafer, Inc, USA). The used device stacks are shown in Fig. 9. Standard PHJ devices have the architecture: ITO/MoO_3_ (10 nm)/DBP (20 nm)/C_70_ (30 nm)/BCP (10 nm)/Ag (100 nm), and inverted PHJ devices: ITO/BCP (0.5 nm)/C_70_ (30 nm)/DBP (20 nm)/MoO_3_ (10 nm)/Ag (100 nm). Standard bulk heterojunction (BHJ) devices were obtained from co-evaporation of DBP and C_70_ materials with 1:1 deposition ratio, having the architecture: ITO/MoO_3_ (10 nm)/DBP:C_70_ (80 nm)/BCP (10 nm)/Ag (100 nm).

Prior to fabrication of the OSC devices, the ITO substrates were cleaned using detergent, acetone, and isopropanol in an ultrasonic water bath (10 min each) and blow-dried with a nitrogen gun. Next, the substrates were treated by plasma cleaning for 20 min, and transferred to a glovebox connected cluster deposition system, which has a base vacuum pressure below 10^−8^ mbar. In this system, the organic material layers, metal oxide interlayers, and metal electrodes were sequentially deposited without breaking vacuum in between the steps. Shadow masks were employed to define the OSC cell area of 10 mm^2^. The deposition rates for the organic materials and Ag top electrode were kept at 0.3 Å/s and 0.5 Å/s, respectively.

The *J-V* characteristics of the OSCs were recorded in ambient conditions using a calibrated 3000 class AAA solar simulator from Abet Technologies Inc., USA connected to a Keithley 2400 source measure unit (Keithley instruments Inc., USA). The J-V characteristics were measured by applying a voltage sweep between $+$ 1 to $-$ 0.25 V using a lamp intensity of 100 mW/cm^2^.

### sEQE measurements

Light from a quartz Halogen lamp (50 W) was chopped at 140 Hz and spectrally dispersed by a monochromator (Cornerstone 260 1 /m, Newport), and then directed onto the mounted sample. The produced current at the short-circuit condition was pre-amplified and converted into a voltage signal. Then the signal was analyzed by a LOCK-IN-amplifier ((7280 DSP, Signal Recovery, Oak Ridge, USA) to measure only the frequency modulated signal. EQE was calculated as the ratio of the photocurrent to flux of the incoming photons (obtained by a calibrated silicon and/or indium-gallium-arsenide photodiode)^61^. The time constant of the LOCK-IN amplifier was set to 1 s to achieve a span spectrum over 5–7 decades, and its amplification was increased to resolve low photocurrents. The sEQE spectra of the devices were analyzed by fitting the low-energy range by employing a home-developed MATLAB code.

### Degradation measurements

For the stability assessment, a climate chamber with 85 °C and 85% RH (in darkness), and a thermal chamber at −40 °C, room humidity, and darkness were used to simulate ISOS-D-3 and ISOS-T-3 degradation protocols, respectively. Devices were placed inside each condition for 24 hours, after which J-V characteristics were recorded using the solar simulator under 1 sun illumination.

### Morphological characterization

AFM images were recorded, using a Veeco Dimension 3100 scanning probe microscope in tapping mode. Vacuum deposition of each sample was performed without breaking the vacuum between the layers, and images were recorded in air directly after fabrication.

### PL quenching measurements

A fluorescence microscope (Nikon Eclipse ME600) with a microscope objective (Nikon E Plan 50 × 0.75 EPL) connected to a Maya2000Pro Spectrometer (from Ocean optics) was used to record the spectra. A mercury short arc lamp having a filtered excitation wavelength centered between 330–380 nm was used as light source. The PL signal was collected with a 10 s integration time, and measurements were repeated after aging the devices as per ISOS test protocols.

### Space-charge limited current (SCLC) measurements on electron-only devices

Electron-only devices were fabricated by sandwiching the C_70_ layer between the respective bottom ETL and top BCP/Ag contact, using the same deposition rates as for solar cell device fabrication. ITO was used as bottom electrodes. J-V characteristics of the devices were recorded by applying a sweeping voltage between $+1$ and ${-}1$ V at reverse bias using a Keithley 2400 source measure unit, directly following fabrication and repeated after aging the devices.

## Supplementary information

Degradation pathways in standard and inverted DBP-C70 based organic solar cells
