# Supplementary material for: Degradation pathways in standard and inverted DBP-C70 based organic solar cells
Source: Sci Rep. 2019 Mar 11;9:4024. doi: 10.1038/s41598-019-40541-6 (PMC6412045; doi:10.1038/s41598-019-40541-6)
Supplement: Supplementary file 1 — Degradation pathways in standard and inverted DBP-C70 based organic solar cells [file 41598_2019_40541_MOESM1_ESM.docx]

**Degradation pathways in standard and inverted DBP-C_70_ based organic solar cells**

Golnaz Sherafatipour^1^, Johannes Benduhn^2^, Bhushan R Patil^1^, Mehrad Ahmadpour^1^, Donato Spoltore^2^, Horst-Günter Rubahn^1^, Koen Vandewal^2, *^, and Morten Madsen^1^

^1^ SDU NanoSYD, Mads Clausen Institute, University of Southern Denmark, Sønderborg, Denmark

^2^ Dresden Integrated Center for Applied Physics and Photonic Materials (IAPP) and Institute for Applied Physics Technische Universität Dresden Nöthnitzer Str. 61, 01187 Dresden, Germany

*Current address: Institute for Materials Research (IMO-IMOMEC), Hasselt University, Wetenschapspark 1, 3590 Diepenbeek, Belgium

Corresponding author: [madsen@mci.sdu.dk](mailto:madsen@mci.sdu.dk)

**Long-term device stability tests**

DBP-C_70_ based organic solar cells with standard configuration were encapsulated using a silicon vacuum sealant (Dow Corning 732) and a glass as the top protection to decrease the effect of extrinsic degradation. Half of the devices were placed inside a thermal chamber providing ISOS-T-3 test condition (-40$℃$ and room humidity-darkness), and the other half were stored in darkness in shelf at room temperature under ISOS-D-1 testing condition. Performances of the devices were recorded regularly using a solar simulator until they reached below their T_20_ value (20% of its initial value). The decay curves of the devices are presented in Figure S.1.

For devices kept under ISOS-T-3 with low temperature, the V_OC_ remains above 80% of its initial value after 3500 hours (almost 145 days). However, for devices kept at room temperature, this point is reached in less than 500 hours (20 days).


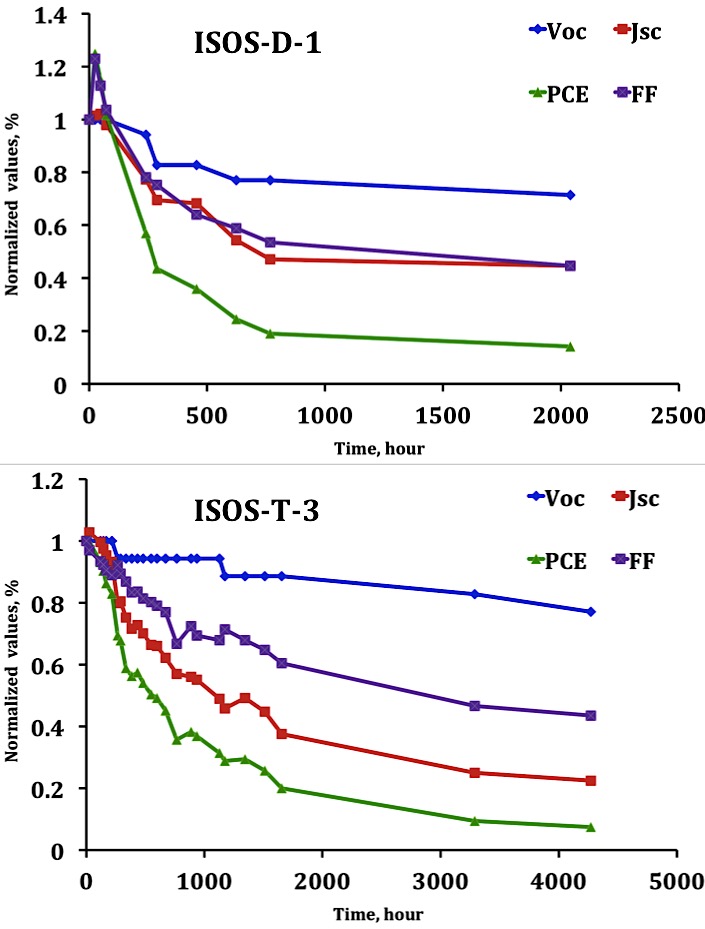


Figure S.1. Lifetime curves of the DBP-C_70_ based organic solar cells with standard PHJ structure, under ISOS-D-1 (top) and ISOS-T-3 (bottom) aging conditions.

**Annealing of bilayer and bulk heterojunction devices**

In order to further study the morphological stability of the standard configuration devices, morphological changes were induced through annealing standard PHJ and BHJ devices at 110 $℃$ for 3 hours. Annealing treatment was performed inside the glovebox to exclude the effect of extrinsic degradation mechanisms. sEQE spectra of the fresh and degraded devices and extracted CT parameters are shown in Figure S.2 and Table S.1. The values are averaged over 7 devices of each type.

Comparing fresh PHJ and BHJ devices, the BHJ shows a higher *f* value, indicating that the BHJ has a higher CT state density due to the increased interface between electron-donating and accepting molecules. However, for both devices, the CT properties *E*_CT_, *λ*, and *f* were not affected by annealing the solar cells, demonstrating stable interface energetics even in morphologically modified active layers.


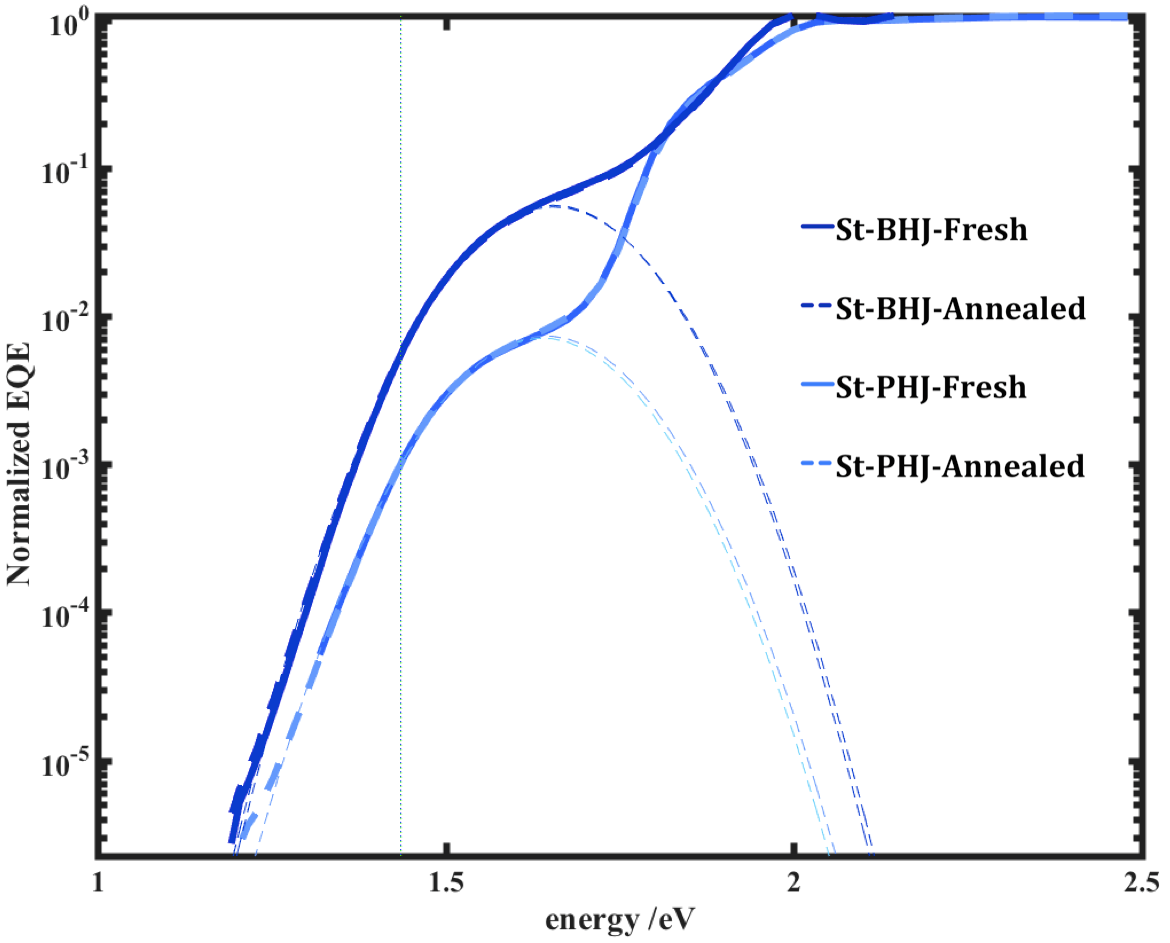


**Figure S.2.** sEQE measurements (solid lines) and Marcus fits (dashed lines) for fresh and annealed devices (110 $℃$ for 3 hours) for solar cells in standard configuration employing either a PHJ or a BHJ. The vertical line at 1.45 eV highlights E_CT_.

Table S.1. Solar cell performance values and CT parameters extracted through fitting the sEQE spectra for fresh and aged devices.

| **Devices** | **V_OC_**  **(V)** | **J_SC_ (mA/cm^2^)** | **PCE**  **(%)** | **FF**  **(%)** | ***f***  **(eV^2^)** | **λ (eV)** | **E_CT_ (eV)** |
| --- | --- | --- | --- | --- | --- | --- | --- |
| **St-BHJ-Fresh** | 0.85 ± 0.04 | 7.49 ± 0.5 | 3.31 ± 0.2 | 51.9 ± 2.9 | 0.011 | 0.17 | 1.45 |
| **St-BHJ-Annealed** | 0.70 ± 0.09 | 6.8 ± 0.5 | 1.87 ± 0.5 | 38.3 ± 7.6 | 0.011 | 0.18 | 1.45 |
| **St-PHJ-Fresh** | 0.85 ± 0.05 | 6.32 ± 0.3 | 3.66 ± 0.2 | 68.0 ± 1.7 | 0.0014 | 0.17 | 1.44 |
| **St-PHJ-Annealed** | 0.87 ± 0.05 | 4.8 ± 0.4 | 2.3 ± 0.2 | 55.9 ± 1.8 | 0.0015 | 0.17 | 1.44 |
